# Partnership work between Public Health and Health Psychology: introduction to a novel training programme

**DOI:** 10.1186/1471-2458-10-692

**Published:** 2010-11-11

**Authors:** Alyssa S Gilinsky, Stephan U Dombrowski, Hannah Dale, Douglas Marks, Clare Robinson, Claire Eades, Despina Ouzounidou

**Affiliations:** 1 Directorate of Public Health, NHS Tayside, Kings Cross Hospital, Dundee, DD3 8EA, UK; 2 Health Services Research Unit, University of Aberdeen, 2nd floor, Health Sciences Research Building, Foresterhill, Aberdeen, AB25 2ZD, Scotland, UK; 3 Department of Psychology, NHS Fife, Stratheden Hospital, Cupar, Fife, KY15 5RR, Scotland, UK; 4 School of Social Sciences, University of the West of Scotland, Paisley, PA1 2BE, Scotland, UK; 5 Pharmacy Services, NHS Forth Valley, Eurohouse, Wellgreen Place, Stirling, FK8 2DJ, Scotland, UK; 6 Directorate of Public Health, Crichton Hall, Lochar North, Bankend Road, Dumfries, DG1 4TG, Scotland, UK

## Abstract

**Background:**

Public health services implement individual, community and population level interventions to change health behaviours, improve healthy life expectancy and reduce health inequalities. Understanding and changing health behaviour is complex. Integrating behaviour change theory and evidence into interventions has the potential to improve services.

**Methods:**

Health Psychologists apply evidence and theories aimed at understanding and changing health behaviour. A Scottish programme is piloting the training of Health Psychologists within NHS contexts to address prominent public health challenges.

**Results:**

This article outlines the details of this novel programme. Two projects are examined to illustrate the potential of partnership working between public health and health psychology.

**Conclusion:**

In order to develop and improve behaviour change interventions and services, public health planners may want to consider developing and using the knowledge and skills of Health Psychologists. Supporting such training within public health contexts is a promising avenue to build critical NHS internal mass to tackle the major public health challenges ahead.

## Introduction

Public health services are increasingly concerned with implementing individual, community and population level interventions to change health behaviours [1]. The shifting of resources towards behaviour change programmes is based on the premise that such interventions will be effective at reducing unhealthy behaviours, thereby improving healthy life expectancy rates and potentially reducing health inequalities. Timely, evidence-based and cost-effective behaviour change interventions could potentially deliver additional benefits to the public in the long-term by enabling resources to be redirected for use elsewhere in the NHS [2].

## Public health and the challenge of behaviour change

Behaviour change interventions are generally complex to design, deliver and evaluate [3]. Public health specialists face multiple challenges when intervening to improve health behaviour, including an insufficient, unclear, unspecific and non-costed evidence base [4]. Interventions explicitly using and incorporating evidence and theory base can have a number of advantages. These advantages include the availability of an explicit theoretical basis for intervention development, which also provides an evaluation framework. This allows programme planners to understand why an intervention had the observed effects, in addition to establishing whether it was effective. This is particularly important in the case of suboptimal intervention effects as it points to potential avenues of intervention improvement. Thus, appropriate use of theory and evidence has the potential to lead to more effective interventions over time [5]. There is potential to improve the application of behaviour change evidence and theory to tackle current public health challenges. In order to improve behaviour change practice, and to advance the science of behaviour change, it is essential that interventions to improve health are systematically developed, evaluated and refined. In this article we argue that Health Psychologists can offer skills to compliment and support public health services in designing, implementing and evaluating interventions to improve health.

## A novel Scottish programme

A novel programme in Scotland is currently piloting the training of Health Psychologists within the NHS. To outline the potential contribution Health Psychologists can make to public health services we provide an introduction to this programme, which to date has been received positively within participating health boards [6]. Below we introduce the programme, preliminary achievements to date and propose potential avenues for further integration of health psychology to work inter-disciplinary with other practitioners to tackle public health issues.

## Health psychology trainees in Public Health

To explore the potential benefits of including health psychology in tackling public health issues, NHS Education for Scotland (NES) recently commissioned a national pilot examining the training of health psychologists within local NHS contexts. To date, NES and local NHS Boards have funded 10 Trainee Health Psychologists (THPs) across Scotland. The core aim of this ongoing pilot is to examine the added value of including Health Psychologists in initiatives targeted at public health challenges with a particular emphasis on reducing health inequalities. Since the beginning of this project in 2008, THPs (following completion of an MSc in Health Psychology) have been engaged in projects and local initiatives to help NHS Boards meet health improvement targets set by the Scottish Government. THP's work consists of two years of supervised practice, provided by at least one experienced practitioner and one Health Psychologist. This model of training provides mutual benefits for both public health and health psychology. NHS public health departments on the one hand can benefit from the contribution of THPs towards nationally and locally identified priorities for health. Health psychology on the other hand can benefit from applying evidence and theory in practical settings by providing THPs with a rich and challenging work environment in which to apply, share and further develop their knowledge and skills. Upon completion THPs have achieved a doctoral level qualification, gained chartership status with the British Psychological Society (BPS) and eligibility for registration with the Health Professions Council (HPC). To date, a wide range of diverse projects have been undertaken by the THPs demonstrating the application of health psychology in areas such as pharmaceutical public health, alcohol use during pregnancy, behaviour change within anticipatory care, breastfeeding, child healthy weight and services for men with cancer. To the authors' knowledge, this represents the first fully funded NHS-based training programme for Health Psychologists in the UK.

### Examples of programme outputs

An interim evaluation of the pilot, based on interviews with THPs (n = 4), supervisors (n = 12) and stakeholders (n = 9) was carried out by NHS Education for Scotland 18 months into the scheme. The evaluation highlighted the unique contribution made by THPs, and reflects their perceived significant contribution [6]. The recognition by supervisors and stakeholders was based primarily on their qualitative evaluation of THPs' performance and outputs around factors including intervention design, delivery of training and individual patient work, but also on evidence from project findings, where available. The two projects outlined below provide examples of the scope, breadth and quality of programme achievements to date.

### Project 1: A behaviour change service for looked after young people

#### The public health problem: health inequalities

Looked after young people (LAYP) are a vulnerable population, who often suffer poorer health than the general population on a range of issues. There is a need for additional support and interventions with this group, which is emphasised by policies.

#### Methodology and health psychology theory

This project commenced with an examination of the research literature, which lacks an evidence base of interventions to improve the sexual health of LAYP. Thus, a needs assessment, to establish the perceived sexual health needs of LAYP, was conducted [7]. Despite difficulties with recruitment, [8], 10 LAYP in Fife were interviewed. Results revealed a need and desire for additional services. Some individuals also wished to discuss sexual health more with workers/carers. There was also a strong desire for services to cover a range of lifestyle issues, not just sexual health.

The findings from the needs assessment were subsequently integrated with health psychology theory for the development of a one-to-one service for LAYP around a range of lifestyle issues. Evidence-based behaviour change techniques were used as a basis for service implementation. Techniques have been linked to a range of theories used in health psychology [9,10], including the Theory of Planned Behaviour, the Health Action Process Approach, Social Cognitive Theory, Information-Behaviour-Motivational Skills model, Operant Conditioning and the Transtheoretical Model. Since some LAYP desired more input from workers/carers, a consultancy service was developed for workers/carers to better support them in working with LAYP around lifestyle issues. Therefore, the service is two-fold: offering intensive one-to-one behaviour change support to LAYP around sexual health, diet, exercise, smoking and non-dependent alcohol and drug use; and consultancy support to their workers and carers regarding addressing healthy lifestyle issues. Young people can be referred or self-refer into the service, which usually sees them for around six one-hour sessions, however this varies according to need. The service aims to build their motivation to engage in healthier behaviours and then build their skills to achieve their goals, aiming to change determinants of behaviour including self-efficacy, outcome expectancies, attitude and planning skills. Carers who access the service have received advice and guidance in working with young people, tailored training, and/or information about health issues.

#### Results/achievements

From March 2009 to February 2010, 40 LAYP self-referred or were referred into the service by a worker/carer. Sexual health (N = 19) was the most commonly discussed lifestyle issue, in addition to smoking (N = 17), healthy eating and physical activity (both N = 12) as well as alcohol (N = 8) and drugs (N = 9). Moreover, 22 workers were offered 36 hours of consultancy.

#### Evaluation and future developments

A feasibility evaluation has now been completed which shows that the intervention is both feasible to deliver in a range of settings, and is highly regarded by LAYP and their workers/carers, despite barriers to its delivery [8]. As this service was a pilot and the evaluation was conducted at an initial stage of service development, it has focussed on the acceptability and feasibility of the service. The acceptability was very high and comments from LAYP and their workers/carers were very positive. Analysis of behaviour change outcomes has shown early promising results based on a small number of individuals. This includes young people quitting smoking, increasing physical activity levels and reporting increased intention to use condoms, actual condom use and access to sexual health services (unpublished data). Further funding has been secured to expand and consolidate the service for a further 14 months following the end of the two-year training post to provide further materials and staff resources (including an additional member of staff - assistant psychologist). This not only allows the service to continue, but also enables further rigorous and extended outcome evaluations to be conducted to examine the effectiveness of the intervention. It is hoped that group interventions may also be developed, particularly for residential settings.

### Project 2: Supporting behaviour change as part of a national anticipatory care programme

#### The public health problem: health inequalities

Decreasing the health gap between the most and least affluent segments of the population constitutes a massive public health challenge cutting across nearly all of public health practice. Health behaviours are one significant target for addressing this discrepancy. As part of the Scottish national anticipatory care programme for cardiovascular health - Keep Well http://www.keepwellscotland.com - supporting health care practitioners' health behaviour change practice has the potential to enhance various elements of the programme, thereby contributing towards reducing health inequalities.

#### Methodology and health psychology theory

A needs assessment was conducted to inform the development of evidence-based and needs-led behaviour change training for health care professionals involved in the programme. The content of the training centred on the use of evidence-based and theory-linked behaviour change techniques [9,10] in relation to delivering cardiovascular health checks. The needs assessment included areas such as perceived training need, perceptions towards delivering behaviour change practice, and barriers & solutions in relation to attending training. This ensured a flexible and individually tailored training programme could be developed. In line with recommendations for changing health behaviour [1], training was based on multiple theories of behaviour and included both motivational (intentions) and volitional (action-orientated) aspects of behaviour change.

#### Results/achievements

Behaviour change training has been offered and rolled out as part of the Keep Well programme. Training so far has included a wide variety of health care professionals such as GPs, pharmacy staff, physiotherapists, practice nurses and practice support staff. The delivery of training has been flexible ranging from one and a half to four hours depending on availability, including repeated training sessions delivered within practice facilities.

#### Evaluation and future

The behaviour change training to date has shown high acceptability and feasibility both in terms of training delivery as well as training content. The training has been delivered in various different settings to differing professions. Evaluative comments towards the training have indicated a high level of satisfaction with the training. In addition, feedback is continuously being used to update, extend and optimise training. In order to complement the face-to-face delivery of training a remote learning resource is currently being developed to allow practices and health care professionals to complete training within their own time and at their own pace. This is practically important for practitioners who find it difficult to physically attend training, due to rurality, resources or time constraints.

## Preliminary conclusions

Public Health could maximise the delivery of appropriate services through the application of health psychology theory and evidence. The novel programme of work described here outlines the feasibility, acceptability and utility of training Health Psychologists within a public health context. Although such promising early work will need to be substantiated through empirical results at later stages, such a programme is likely to be able to be replicable in other areas of the UK. As part of a multidisciplinary public health team, Health Psychologists complement the range and scope of skills needed, drawing on theories and techniques that are sometimes not fully integrated in applied settings. Moreover, through the implementation of evaluation procedures, elements linked to intervention effectiveness can be identified and interventions can be optimised. At times when resources in applied NHS settings are sparse, these skills are vital not only to improve services, but also to ensure that cost effective programmes are implemented. The future capacity of the NHS to deliver evidence-based public health interventions is predicated on an appropriately skilled workforce. Providing training for Health Psychologists within a public health context has potential benefits for NHS services as demonstrated in the pilot scheme in Scotland.

## Competing interests

The authors declare that they have no competing interests.

## Authors' contributions

AG co-ordinated the drafting of the manuscript in full, however, DM contributed extensively to the original draft. The projects discussed in detail in this article represent the work of HD and SD. CR, DO and CE helped to draft the manuscript. All authors read and approved the final manuscript.

## Authors' information

All of the authors are current or past Trainee Health Psychologists working in NHS contexts to tackle present day public health challenges.

## Pre-publication history

The pre-publication history for this paper can be accessed here:

http://www.biomedcentral.com/1471-2458/10/692/prepub
